# A mutation degree model for the identification of transcriptional regulatory elements

**DOI:** 10.1186/1471-2105-12-262

**Published:** 2011-06-27

**Authors:** Changqing Zhang, Jin Wang, Xu Hua, Jinggui Fang, Huaiqiu Zhu, Xiang Gao

**Affiliations:** 1 State Key Laboratory of Pharmaceutical Biotechnology, School of Life Science, Nanjing University, Nanjing 210093, China; 2 Model Animal Research Center, Nanjing University, Nanjing 210093, China; 3 Department of Biomedical Engineering, and Center for Theoretical Biology, Peking University, Beijing 100871, China; 4 College of Horticulture, Jinling Institute of Technology, Nanjing 210038, China; 5 College of Horticulture, Nanjing agricultural university, Nanjing 210095, China

## Abstract

**Background:**

Current approaches for identifying transcriptional regulatory elements are mainly via the combination of two properties, the evolutionary conservation and the overrepresentation of functional elements in the promoters of co-regulated genes. Despite the development of many motif detection algorithms, the discovery of conserved motifs in a wide range of phylogenetically related promoters is still a challenge, especially for the short motifs embedded in distantly related gene promoters or very closely related promoters, or in the situation that there are not enough orthologous genes available.

**Results:**

A mutation degree model is proposed and a new word counting method is developed for the identification of transcriptional regulatory elements from a set of co-expressed genes. The new method comprises two parts: 1) identifying overrepresented oligo-nucleotides in promoters of co-expressed genes, 2) estimating the conservation of the oligo-nucleotides in promoters of phylogenetically related genes by the mutation degree model. Compared with the performance of other algorithms, our method shows the advantages of low false positive rate and higher specificity, especially the robustness to noisy data. Applying the method to co-expressed gene sets from Arabidopsis, most of known *cis*-elements were successfully detected. The tool and example are available at http://mcube.nju.edu.cn/jwang/lab/soft/ocw/OCW.html.

**Conclusions:**

The mutation degree model proposed in this paper is adapted to phylogenetic data of different qualities, and to a wide range of evolutionary distances. The new word-counting method based on this model has the advantage of better performance in detecting short sequence of *cis*-elements from co-expressed genes of eukaryotes and is robust to less complete phylogenetic data.

## Background

Transcriptional regulation is a major step to determine the spatial and temporal activities of genes in eukaryotes. Various stimuli, whether external or internal, activate transcription factors. Then the transcription factors initiate or repress the transcription of target genes by binding to the specific sites (named transcription factor binding sites, TFBSs or *cis*-elements) embedded in promoter sequences. Therefore, identifying these functional regulatory elements from gene promoters seems to be a promising way to decipher how the gene regulatory network is orchestrated [1,2]. With the availability of huge genomic data and other omics data, as well as the high performance computers, computational strategy has shown the great potential in the discovery and functional characterization of *cis*-elements in many biological aspects [3].

The methods based on the principle of over-representation identify *cis*-elements mainly by detecting the motifs that occur more frequently in a set of promoters of genes that may be expression-related or biological process related. While this class of algorithms is successful in identifying many well-characterized *cis*-elements, they are still limited in determining the true elements through which a specific set of genes are activated in certain biological processes, i.e., this type of methods has the problem of high false positive rate. Because, a piece of oligo-nucleotide (also known as a word in the field of sequence analysis) presented in most of the promoters of related genes does not necessarily mean that the genes are regulated via this short stretch of sequence. However, if the stretch is also conserved in the promoters of phylogenetically related genes, then it is more probable that the oligo-nucleotide is a functional element. Thus, one way of improving the reliability of prediction algorithms is to introduce the property of phylogenetic conservation.

So far, there are mainly two classes of phylogeny-based methods of *cis*-element identification. One of them is based on sequence comparison in an assumption that the functional elements are more conserved than their flanking sequences. So the most conserved segments are predicted as the functional elements through global or local sequence alignments and with the help of phylogenetic trees in some tools [4,5]. The disadvantage of this class of methods is that the prediction is, to a large extent, dependent on whether the user can retrieve a set of phylogenetic related genes with a proper evolutionary distance. For the closely related species, the promoter sequences are too similar to distinguish the regulatory elements. In contrast, some short functional elements in evolutionarily distant sequences are usually not well pre-aligned into the local multiple alignment, so they would be easily missed by the phylogenetic models.

Another class of prediction methods that circumvent the problem of sequence alignment assumed that not all the *cis*-elements are aligned to the most conserved regions through sequence comparison. Instead, they directly identify the motifs from the orthologous promoters based on a series of regulatory element features including the sequence conservation, over-representation and the conserved distance between elements etc. These algorithms are not so restricted to the evolutionary distance of orthologous genes and thus are more adaptive to divergent biological problems. However, there are also many shortcomings accompanied to this type of prediction methods. Some of the tools only consider the phylogenetic relations between two species, for example, orthoMEME [6]. Others even equally treated the sequences of different evolutionary distances [7]. Many of the methods that integrate phylogenetic relations are still confronting the difficulty of providing the reliable evolutionary information. The phylogenetic trees requested by PhyloGibbs [8] and PhyME [9], or the substitution value requested by EmnEM [10] and weederH [11] for producing the phylogenetic relations are more or less experiential and frequently result in errors in the prediction results. The situation becomes even worse when the orthologous gene set is not well prepared.

So it seems that the quality of phylogenetic data comprises one of the determinants in the computational identification of functional elements from evolutionary related gene promoters. For vertebrates, yeasts and fruit flies, there are a lot of genome sequences available. But, for plants, the sequencing and annotation of genomes lag far behind [12]. As a matter of fact, even though there are enough genome data available, it is not very easy to collect a reliable set of orthologous genes in some cases. This kind of restrictions limits the practical application of computational tools in various biological problems. Therefore, new models dealing with the evolutionary relations for the detection of *cis*-elements from phylogenetic data are needed. In addition, there are many practical cases in which users prefer to first work on the most reliable predictions of gene regulation relation in wet lab. In this case, decreasing the false positive rate in predicting functional elements from a set of co-expressed genes is critical.

To identify the potential functional motifs from diversiform phylogenetic data, we proposed a mutation degree model, which incorporates word-counting algorithm to detect the overrepresented oligo-nucleotides in a set of phylogenetical sequences. By combining the new model with the over-representation property of functional element in co-expressed gene sets, a new tool named OCW (Over-represented and Conserved Word) was developed to identify *cis*-elements from co-expressed gene sets. The feasibility of the new method was evaluated on synthetic data with different identity, co-expressed gene data and noisy phylogenetic data with different number of random sequences.

## Results

### Evaluation of OCW on synthetic data with different evolutionary distances

To benchmark OCW and other tools, 4 sets of gene sequences are required for one test: co-expressed genes and their background genes, orthologs of the co-expressed genes and their background sequence. Here, we first generate the sequences of co-expressed genes and their background genes based on the information that 25% probability for A, C, G, T, 1000 bp long for co-expressed genes and their background genes, at least one instance of the 6 bp motif which allows 1 bp mutation was randomly planted into the co-expressed gene. The number of co-expressed genes is 7 and that of the background sequences is 210 (thirty times that of the co-expressed genes). Next, we generate the orthologs of the co-expressed genes and their corresponding neutral sequence. First, we assigned a certain mutation extension, like <20%, to co-expressed genes, and then mutated each sequence of the dataset for 5 times at random to created a set of orthologous sequences with 5 members for each co-expressed genes. After then, we further created the neutral promoter by using the base composition of each member of the orthologs. Here, the length of neutral promoter was 5000 bp (five times that of the ortholog), and 4 mutation levels, <20%, <40%, <60%, and <80%, were respectively used for each set of co-expressed genes. As a result, 60 groups of synthetic test data representing 15 repeats and 4 mutated levels were generated.

To evaluate the performance of the new method, we applied OCW, AlignACE[13], GLAM2[14], Weeder[15], PhyloGibbs[8], PhyloCon[7] and WeederH[11] to the synthetic data. Following Tompa [16], we used the nucleotide-level sensitivity (nSN), specificity (nSP) and positive predictive value (nPPV) to evaluate the performance. Where, the nPPV shows the nucleotide fraction of predicted known sites out of the total positive predictions. The nSN shows the nucleotide fraction of predicted known sites out of the actual known sites. And the nSP represents the nucleotide fraction of predicted non-site over the actual non-sites.

**Figure 1:**
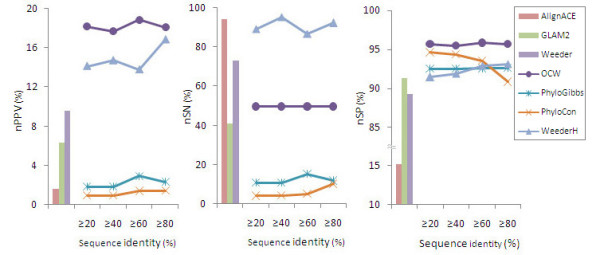
**Performance comparisons of different tools on simulated data**. The predictions shown in histogram are from AlignACE, GLAM2 and Weeder. These three tools are based on over-represented word detection. The predictions shown in line chart are from OCW, PhyloGibbs, PhyloCon, and WeederH, which introduced phylogenetic information in the algorithms. The extent of convergence of artificial orthologous sequences used in these tools is represented by the sequence identity.

As showed in Figure 1, the assessment value nPPV of OCW is around 18%, which is higher than that of all the other six tools. This means OCW has a higher effect in reducing the false-positive rate of motif prediction. The value of nSN for OCW is around 50%, which is lower than the three tools, AlignACE, Weeder and WeederH, while the nSP value of 95% is higher than all the other tools under the assessment. This result indicates that OCW has a stronger capacity of non-site discrimination. Therefore, OCW has gone beyond the other 6 tools in positive predictive value and in specificity, which provide a new choice for user-expected lower false-positive rate or higher exclusion of non-site sequences.

What is also shown in Figure 1 is the assay of the ability of robustness of the prediction methods to the divergence of orthologous sequences. When the identity between the sequences was increased from ≥20% to ≥80%, they show little change on the values, nPPV, nSN, and nSP. Especially, the values from OCW are more stable than others, which indicate that OCW is more tolerant to the wide range of sequence evolutionary distances.

OCW and WeederH could be categorized as the tool of word enumeration algorithm, and PhyloGibbs and PhyloCon as the heuristic algorithms. Comparing the performance of the two types of tools, enumeration algorithm is better than that of the heuristic algorithms, suggesting that enumeration model has superiority in detecting the functional motifs from co-expressed and orthologous sequences, which agrees to the previous opinion [17].

### Performance of OCW in detecting functional elements from co-expressed genes

To evaluate the feasibility of OCW on biological data, we applied it on 7 sets of co-expressed genes from Arabidopsis. They were obtained from literatures [18-22] that are listed in Table 1. The background sequences are from the promoters of Arabidopsis genome excluding the co-expressed genes. The phylogenetic promoters were retrieved from the plant database of DoOP (version 1.5) [23]. Their background sequences, i.e. the neutral sequences, or referred as a set of phylogenetically unrelated promoters, were built by sampling 100 promoters randomly from its corresponding genome.

**Table 1:** The functional elements detected by 7 tools*

Dataset	Binding site	OCW	WeederH	PhyloGibbs	PhyloCon	AlignACE	GLAM2	Weeder	**Ref**.
1	ABRE(ACGTGKC)	+	+	+	-	+	+	+	[18]
	DRE(TACCGACAT)	+	-	-	+	+	+	-	[18]
2	ARF(TGTCTC)	+	+	+	+	+	+	+	[19]
3	ARF(TGTCTC)	+	+	+	+	+	-	+	[19]
4	XBP1BS/P-UPRE/ERSEI(CCACGTCAT)	+	+	+	+	+	-	+	[20]
	P-UPRE/ERSEI(ATTGGN9CCACG)	+	+	+	+	+	+	+	[20]
5	G-box(CACGTG)	-	+	+	+	+	+	+	[21]
6	SAUR(CATATG)	+	+	-	-	-	+	+	[22]
7	ABRE3(CAACGTG)	+	+	+	-	-	+	-	[22]
	extA(AACGTGT)	+	+	-	-	-	+	-	[22]

* Only those elements already reported in literatures are listed. '+' indicates successful detection, '-' means failed detection.

As shown in Table 1, OCW performed well and successfully detected 9 *cis-*elements reported in literatures for 6 of 7 sets of co-expressed genes. Compared with the other six tools, WeederH, PhyloGibbs, PhyloCon, AlignACE, GLAM2 and Weeder, OCW detects much more known sites. In consistence with the evaluation results from synthetic data shown in Figure 1, this result further indicates that OCW is better than other tools, against true biological data.

The element G-box in dataset 5 was not detected by OCW because it did not pass the Fisher's exact test at the second step of OCW method.

### Performance on noisy data

To further measure the performance of OCW against noisy data, i.e. data with unreliable phylogenetic genes, we artificially introduced several sequences that were randomly retrieved from genomes into the phylogenetic promoter set. Here, the 25 co-regulated genes including *STE3 *and *MFA2 *through the motifs MCM1 and MATalpha2 are from *S. cerevisiae *genome. The background sequences are 196 and picked from *S. cerevisiae*. The orthologous promoter sets of *STE3 *and *MFA2 *were retrieved from genomes *S. cerevisiae *and *S. castellii*, and their background sequences are merged sequences of 30 non orthologous promoter randomly retrieved from genomes *S. cerevisiae *and *S. castellii*, where the two genes *STE3 *and *MFA2 *should be excluded. The noisy promoters were generated by sampling promoters randomly from the *S. cerevisiae *and *S. castellii *genomes. In the tests, 2, 4, 6 and 8 noisy promoters were jammed into the phylogenetic promoter sets of *STE3 *and *MFA2*, respectively, which was repeated for 5 times to reduce the sampling error.

**Figure 2:**
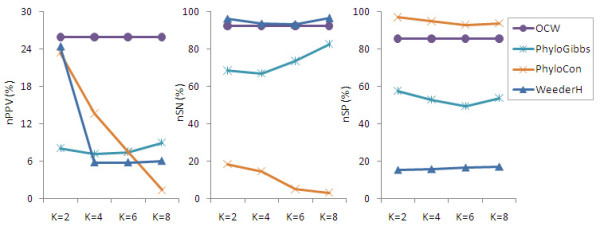
**Performance of OCW, PhyloCon, PhyloGibbs and WeederH on noisy data**. The extent of noise was adjusted by introducing an increasing number (k) of random promoters into the phylogenetic sets.

Since the tools of AlignACE, GLAM2, and Weeder were designed only to co-expressed genes, we only benchmark the performance of tools, PhyloGibbs, PhyloCon, WeederH and OCW here. Figure 2 shows the result. Compared with the implementations of PhyloGibbs, PhyloCon and WeederH, OCW shows little variation with the increasing number of noisy sequences. While PhyloCon shows a sharp decrease in the ability of detecting known elements (see nSN and nPPV in Figure 2) as the noisy data were increased, although it keeps a good specificity (nSP) during this process. PhyloGibbs shows an increasing sensitivity to the introduction of noisy data, but the specificity decreases significantly, although the nPPV value tends to be stable. This result showed that OCW has a greater tolerance to noisy data.

## Discussion

One of the biggest challenges in the era of systems biology is the discovery of complex gene regulatory networks [24]. To seek the gene regulatory relations, the detection of transcription regulatory elements that control gene expression is regarded as a fundamental task. To decrease the high false-positive rate of many motif-discovery algorithms [25], conservation property of functional elements were introduced by multiple sequence alignment or other strategies that utilize phylogenetic information derived from orthologous sequences. This class of algorithms normally performs well on a set of phylogenetic genes with appropriate diverging time [26]. Unfortunately, in most of the practical cases, especially in plant kingdom, to collect a reliable set of orthologous genes is often difficult. We tried to tackle this problem by the mutation degree model proposed in this paper. We firstly detect the over-represented words in a co-expressed gene set, and then, evaluate the conservation extent of these words in a phylogenetically related promoter set by applying our new mutation degree model. We named the whole approach as the OCW method.

Based on the evaluation results, we found that OCW showed two advantages over the current methods, the lower rate of false-positives and the higher ability of noise tolerance. Both of the advantages are beneficial to the identification of *cis*-elements in practical cases. For example, many users may hope to get a reliable prediction of the functional elements from a set of co-expressed genes for further experimental verification. In this case, decreasing the false positive rate of the prediction is critical. Normally, it is not very easy to construct a set of phylogenetic promoter set of high quality, especially in plant kingdom. So, the tolerance of the tools to noisy data is of special importance in dealing with a wide range of practical biological problems.

Resulted from the current difficulties in identifying orthologous gene set, many non-orthologous genes are often mistaken for orthologous genes, which significantly affects the accuracy of *cis*-element prediction. In our mutation degree model, a step of pre-alignment of promoters has been introduced to obtain the mutation degree allowed for the enumerated words. Meanwhile, this process also has the effect of ruling out the false homologous promoters. As showing in Figure 2, the performance of OCW against the increasing number of noisy sequences is much better than the other tools.

Reduction of false-positive rate has remained a big problem in the computational identification of transcriptional regulatory elements. Compared to the popular tools currently used in the discovery of functional motifs, OCW shows the best performance of an nPPV of about 18% (Figure 1) or 26% (Figure 2). Yet, this value is not good enough. There is still a long way to go in improving the prediction method. A similar conclusion was drawn from a previous evaluation. In Tompa's experiment on assessing the tools of finding transcription factor binding sites [16], nPPV remained under 15% in most of the cases.

Despite of the advantages of OCW shown above, there are still some limitations, for example, the relatively low sensitivity of about 49% as shown in Figure 1. This is mainly resulted from the simple application of Fisher's test in the production of overrepresented oligo-nucleotides out of the co-expressed gene sets. Further study will focus on the design of new models for the detection of overrepresented words and to optimize OCW to improve the sensitivity. Besides, OCW does not consider the interactive relations of *cis*-elements, like that of the *cis-*module, but only counts the number of sites. What should also be noted is that OCW was designed to identify the short motifs for eukaryotic genes. All the assessments in this study were performed on short motifs.

In developing the tool OCW for identifying functional elements from co-expressed gene sets and orthologouse gene sets, we mainly focused on the reduction of false positives and the elevation of tolerance to noise data. By artificially introducing the unrelated sequences into the phylogenetically relevant promoter sets, we showed the robustness of OCW to the orthologous sequence set of low quality. The improvement in decreasing the false positive rate is illustrated by the assessment of a couple of tools on synthetic data. The feasibility of OCW in identifying the functional motifs was shown by applying the tool to several sets of co-expression genes of Arabidopsis. OCW found the most number of know sites. The results from this study also support the previous suggestion that enumeration algorithm has some superiorities over the heuristic algorithms in detecting the functional motifs from co-expressed and orthologous sequences.

## Conclusions

We present a mutation degree model to deal with the sequence variation of functional element in different species. Our new model is adapted to phylogenetic data of different qualities, and to a wide range of evolutionary distances. Using this model, we developed a new word-counting method for identifying short motifs of transcriptional regulatory elements from a set of co-expressed genes, by utilizing a group of phylogenetic related gene promoters. Compared with other motif detection programs, our method is more effective and more adaptive to less complete phylogenetic data or noisy data. Thus, this model will find a wider application in gene expression analysis, especially in exploring new regulation mechanisms in species that have not been well studied.

## Methods

### The mutation degree model

We assume that transcription regulatory elements are conserved in a set of phylogenetically related promoters, as these sequences may share the same regulatory mechanism. So they should show a higher occurrence frequency among these phylogenetically related sequences. We score the extent of over-representation of a motif to infer its conservation by a word-counting algorithm [27], i.e. *S *in formula (1) could be regarded as an approximation of conservation score of a motif in this promoter set.(1)

Where *AO *refers to the actual occurrence of an oligo-nucleotide in the phylogenetically related promoter set, *EO *is the expected occurrence, and *k *is a correction factor.

To enhance the signal-to-noise ratio, two corrections are introduced as *k_1 _*and *k_2_*. The former is defined to correct the bias of neutral promoter set, in which the members have no phylogenetic relationship.(2)

Where *EO' *is the expected occurrence of an oligo-nucleotide in the set of phylogenetically unrelated promoters; *AO' *is the corresponding actual occurrence.

Another correction, *k_2_*, is for bias of neutral oligo-nucleotide that is assumed to be non functional in a promoter.(3)

Where *EO'' *and *AO'' *are respectively the expected and actual occurrences of a neutral oligo-nucleotide presented in the phylogenetically related promoter set, *n *is the total number of all possible oligo-nucleotides that are presented in the same promoter and have the same length as the one under study.

In a long random DNA sequence of composition *P_a_*, *P_c_*, *P_g_*, *P_t_*, the expected occurrence probability of an oligo-nucleotide *M *of length *L_m _*could be estimated as [28,29], where *P_i_*∈{ *P_a_*, *P_c_*, *P_g_*, *P_t _*}. So the total occurrence of the motif in all the *N *sequences, taking into account both strands, is calculated as(4)

Where *L_p _*is the length of promoter sequence.

If an oligo-nucleotide is over-represented in a set of phylogenetically related sequences, the ratio of actual occurrence to expected occurrence should be larger than that in the neutral sequences set. Similarly, the ratio should be larger than that of the neutral oligo-nucleotide with the same length. Therefore, we regard an oligo-nucleotide as conserved only when its *S *values are larger than 1 after both corrections.

**Figure 3:**
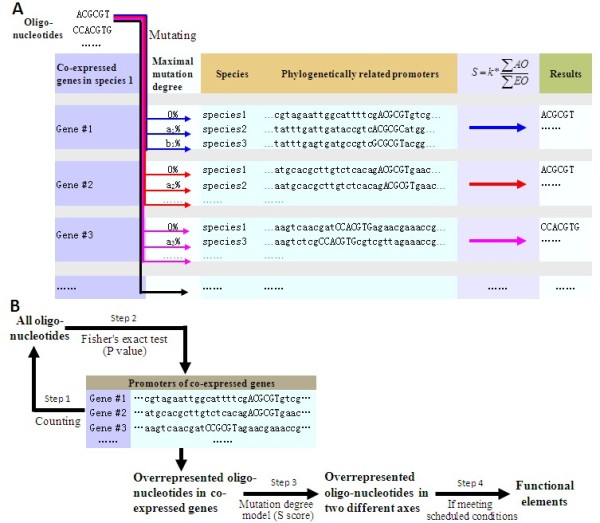
**Illustrations for mutation degree model and OCW method. (A) Illustration of the mutation degree model**. The phylogenetic promoter sequences of Gene#1, Gene#2 and Gene#3 *etc*. are highlighted in light blue. Mutation degrees between the promoter of species1 and its phylogenetic related promoters are denoted as a_1_%, b_1_%, etc. The data in the result column is only for demonstration. The co-expressed gene set highlighted in lavender belongs to Species1. **(B) Flow chart of OCW**. Step 1: All oligo-nucleotides presented in co-expressed genes are enumerated; Step 2: Fisher's exact test of the over-representation significance of the enumerated oligo-nucleotides; Step 3: Calculation of the conservation score of the elements resulted from step 2, the elements with S>1 are reported; Step 4: Reporting functional elements that meet the criteria assigned by user.

The mutation of functional element in different species is further considered by introducing the mutation degree as illustrated in Figure 3(A). Compared with those models based on phylogenetic tree, it does not use phylogenetic tree. Conversely, based on the fact that *cis*-elements located in the same promoter, whether long or short, and in either strand, usually functions in a combinatorial and cooperative manner [30], we assume that they are under the same evolutionary selection pressure and have a similar mutation degree. Here a local sequence alignment tool for comparing two sequences is used [31] to obtain the conserved block with maximal mutation. Then the mutation degree, *μ*, between two sequences is estimated from this region. Where, *μ *may vary in different pairs between the reference promoter and its orthologs, i.e., the values a_1_%, b_1_%, etc. in Figure 3A may be different.

We assign the number of mutational instances of an element as *f(μ)*. The actual occurrences of all the mutational instances of an element in a promoter were summarized. So, the model measuring the conservation extent of an oligo-nucleotide in a set of phylogenetically related promoters can be modified as formula (5).(5)

Where *AO(inst._j_) *indicates the actual occurrence of mutational instance *j *of an oligo-nucleotide in promoter *i *(*j *= *1*,..., *f(μ)*; *i *= *1*,..., *N*, *N *is the number of promoters included in a phylogenetically related gene set.). *EO(inst._j_) *indicates the corresponding expected occurrence of instance *j*.

### The method OCW for identifying transcription regulatory motifs

We joined the new mutation degree model to the method of identifying overrepresented oligo-nucleotide in set of co-expressed genes and proposed a new method, OCW http://mcube.nju.edu.cn/jwang/lab/soft/ocw/OCW.html, for the prediction of transcriptional regulation elements from a set of co-expressed genes.

Figure 3(B) shows the flow chart of this tool. Where, OCW begins with enumerating all the oligo-nucleotides of e.g. 6-10 bp presented in the promoters of co-expressed gene set and examines their statistical significance of over-representation through Fisher's exact test as follows:

Where *n_1 _*denotes the number of co-expressed promoters containing an element, *n_2 _*is the number of co-expressed promoters that do not contain this element; *m_1 _*is the number of background promoters containing the element, *m_2 _*is the number of background promoters not containing the element.

The resulting over-represented elements in the co-expressed gene set are then further evaluated by conservation score or the overrepresentation score in each of the phylogenetically related promoter set by applying the mutation degree model. Finally, the elements overrepresented both in co-expressed genes and in phylogenetically related promoters are determined.

### Implementing the tools

#### On synthetic data

Seven established tools, AlignACE 3.0, GLAM2, Weeder, PhyloGibbs, PhyloCon, WeederH and OCW were applied. According to the categories of these tools, the following rules were adopted in the implementations. (1) For AlignACE, GLAM2, and Weeder, since they detect motifs over-represented in co-regulated genes while none of them take into account the phylogentic information, we only provided co-expressed gene data to these tools. The parameters were all default except that the GC content is 0.50 for AlignACE, 'yeast' was chosen for the selected check-box and the selected organism for Weeder. All of these tools were obtained from their website. (2) PhyloGibbs, PhyloCon and WeederH all use the phylogentic information and fit to the category of finding motifs in sets of orthologs. We organized the synthetic data into the orthologous set according to each co-expressed gene and then provided the tools with these data. For PhyloGibbs, we also pre-aligned the orthologs by using DIALIGN program. They were all running at local machine, and implemented with the default parameters, except that the 'number of standard deviations' was set to 1 for PhyloCon, and the motif length was set to 6 bp for PhyloGibbs, and the selected organism, yeast, for WeederH. (3) For OCW, the P value used in Fisher's exact test is 0.01. The maximum mutation degree was generated as follows: a local sequence alignment tool, BL2SEQ, was used to align the reference promoter to each of its phylogenetically related members, and then, based on the minimum similarity (*min_similarity_*) produced from each of their alignment, we use the function *μ *= 1-*min_similarity _*to get the maximum mutation degree. The criterions of *S *are over 1.1 in both corrections.

#### On biological data

(1) For AlignACE, GLAM2, and Weeder, the parameters were all taken as the default except that the selected check-box of looking for motifs in both strands, the selected check-box of thinking a motif might appear more than once in a single sequence, and the selected organism, arabidopsis, for Weeder. (2) For PhyloGibbs, PhyloCon and WeederH, the implementation of PhyloGibbs and PhyloCon followed the same rules as that on synthetic data, except that the motif length was set to 8-11 bp in PhyloGibbs, and the selected organism, Arabidopsis, for WeederH. (3) OCW followed the same rules as that on synthetic data except that the motif length is set to length of true motif.

#### On noisy data

Running PhyloGibbs, PhyloCon, and OCW followed the rules as that on synthetic data, except that the motif length was set to 8-11 bp for PhyloGibbs.

### Performance evaluation

Following Tompa [16], we used the nucleotide-level sensitivity (nSN), specificity (nSP) and positive predictive value (nPPV) to evaluate the performance of different motif detection algorithms. We define nTP as the number of nucleotide positions both in known motifs and in predicted motifs. nFN is the number of positions in known motifs but not in predicted motifs. nFP is the number of positions not in known motifs but in predicted motifs. nTN is the number of positions in neither known motifs nor in predicted motifs. Sensitivity is defined as nSN = nTP/(nTP+nFN), specificity is defined as nSP = nTN/(nTN+nFP), and positive predictive value is defined as nPPV = nTP/(nTP+nFP).

## Competing interests

The authors declare that they have no competing interests.

## Authors' contributions

CQZ and JW designed the algorithm, measured tools in biological data and noisy data, and drafted the manuscript. XH measured tools in synthetic data and benchmarked with other programs. JGF critically read the draft and contributed to the design of the algorithm. HQZ and XG conceived of the study, coordinated the work and helped to draft the manuscript. All authors read and approved the final manuscript.
